# Absorption of Lead and Mercury in Dominant Aquatic Macrophytes of Balili River and Its Implication to Phytoremediation of Water Bodies

**DOI:** 10.21315/tlsr2020.31.2.2

**Published:** 2020-08-06

**Authors:** Jones T. Napaldet, Inocencio E. Buot

**Affiliations:** 1 Biology Department, Benguet State University, Km. 6, La Trinidad, Benguet, 2601 Philippines; 2 Institute of Biological Sciences, University of the Philippines Los Baños, Philippines

**Keywords:** Mercury, Lead, Balili River, Aquatic Macrophytes

## Abstract

In the Philippines, phytoremediation studies on heavy metals are mainly concentrated in mining areas amidst several reports of heavy metal contamination even in non-mining sites in various parts of the country. Such was the case Balili River which was reportedly contaminated with mercury (Hg) and lead (Pb). Aquatic macrophytes growing in the river could offer the solution to this problem via phytoremediation. Thus, this study was conceptualised to determine the uptake of Hg and Pb in selected dominant macrophytes of the river namely *Amaranthus spinosus*, *Eleusine indica* and *Pennisetum purpureum*. Soil, water and plant samples gathered from the study sites were submitted to Department of Science and Technology-Cordillera Administrative Region (DOST-CAR) laboratory for Hg and Pb determination. Soil and wastewater of Balili River were found contaminated with Pb but not with Hg. The soil recorded higher Hg concentration than water while Pb concentrations did not differ between the two media. The aquatic macrophytes in the study registered consistently higher Hg and Pb in their shoots > roots but differed in their capacities and distribution in the shoot organs. Hg and Pb accumulation was significantly (*p* = 0.00) higher in stem of *P. purpureum* while in *E. indica*, leaf had the highest accumulation, though not statistically significant (*p* = 0.09). For *A. spinosus*, Hg was highest in its leaf while Pb in stem, though not statistically significant (*p* = 0.06). Among the three macrophytes, *P. purpureum* showed the highest potential for Hg uptake and translocation and for Pb uptake. On the other hand, the highest Pb internal transfer was recorded in *E. indica*. These results contradict initial findings that Pb is mostly accumulated in plant roots with minimal shoot translocation. Also, these results show that local macrophytes in Balili River, even if obnoxious weeds, are ecologically important and could be used for phytoremediation of local rivers that are recipient of small-scale mine tailings.

**Highlights**

- The Hg and Pb lead accumulation and uptake in *Amaranthus spinosus, Eleusine indica* and *Pennisetum purpureum* in Balili River, Benguet, Philippines is reported.
- *Pennisetum purpureum* showed the highest potential for Hg uptake, Hg translocation and Pb uptake while *E. indica* had the highest Pb translocation.
- Our results contradict initial findings that lead is mostly accumulated in plant roots with minimal shoot translocation.
- Local macrophytes in Balili River, even if obnoxious weeds, are ecologically important and could be used for phytoremediation of local rivers that are recipient of small-scale mine tailings.

## INTRODUCTION

Heavy metal contamination of the environment is of great concern in today’s society due to its prevalence in global scale, the high degree of difficulty in its clean-up and its high threat to human health (Adriano *et al*. 1995; Bini 2010). Conventional physico-chemical clean-up of heavy metal is often cost- and technology-intensive (e.g. in the U.S. up to USD250 m^−3^ soil) and is mostly not for developing countries like the Philippines. Thus, alternative options like phytoremediation are highly sought. In the previous decades, attention has been focused to plants as tools to clean up metal-contaminated areas with particular emphasis on the ability of plants to accumulate high heavy metal concentrations (up to 100 times the normal concentration) in their aerial parts (Baker 1981). Because it is a natural process, phytoremediation is now an emerging potential effective technology in reclaiming contaminated areas because of its cost effectiveness, aesthetic advantage, “green clean” approach and long-term applicability to a wide range of contaminants (Tulod *et al*. 2012).

In the Philippines, several experts and authorities recognised the potentials of phytoremediation dubbing it as “green technology” (Paz-Alberto & Sigua 2013; See 2014). However, very few actual efforts were being conducted to realise these potentials amidst the prevalence of heavy metal contamination in different parts of the country. Usually, phytoremediation studies in the country were concentrated in mining areas (Claveria *et al*. 2010), neglecting or assigning very little attention to other areas that are most likely contaminated by same heavy metals.

One such area is Balili River, an important water resource of Baguio City and La Trinidad, Benguet. Regular water quality monitoring by Department of Environment and Natural Resources-Cordillera Administrative Region (DENR-CAR) revealed that the river is consistently contaminated with lead (Pb). There were also spot-reports that it is contaminated with mercury (Hg). There are several possible sources of Pb in Balili River such as paints (from old buildings or discarded residues), furniture and car shops, vehicular emissions that settles on the water or have been carried by run-off, and many others. On the other hand, Hg in the river is most likely coming from clean up of mine tailings and Hg-based gold recovery activities.

Aquatic macrophytes growing in Balili River could offer the most viable solution to its Pb and Hg contamination. Plants are categorised into three groups according to their strategies for growing on metal-contaminated soils; metal excluders, indicators and accumulators or hyperaccumulators (Baker & Walker 1990). Metal excluders are plants which effectively limit the levels of heavy metal translocation within them and maintain relatively low levels in their shoot over a wide range of soil levels; however, they can still contain large amounts of metals in their roots. Metal indicators are plants that accumulate metals in their aboveground tissues and the metal levels in the tissues of these plants generally reflect metal levels in the soil, however, continued uptake of heavy metals cause these plants to die-off. Metal accumulators (hyperaccumulators) are plant species that concentrate metals in their above-ground tissues to levels far exceeding those present in the soil or in the non-accumulating species growing nearby. These plants are capable of extracting heavy metals from soils and concentrate them in their shoots.

With this background information, it was clear that before we make plans for phytoremediation of Balili River, there was a need to first characterise the response of aquatic macrophytes in the area to heavy metals. And this exactly was what the study aimed to uncover. The metal concentration values obtained were used to calculate the Bioconcentration Factor (BCF) and Translocation or Transfer Factor (TF), which were used to quantitatively express metal tolerance or avoidance.

## MATERIALS AND METHODS

### Study Area

**Figure 1:**
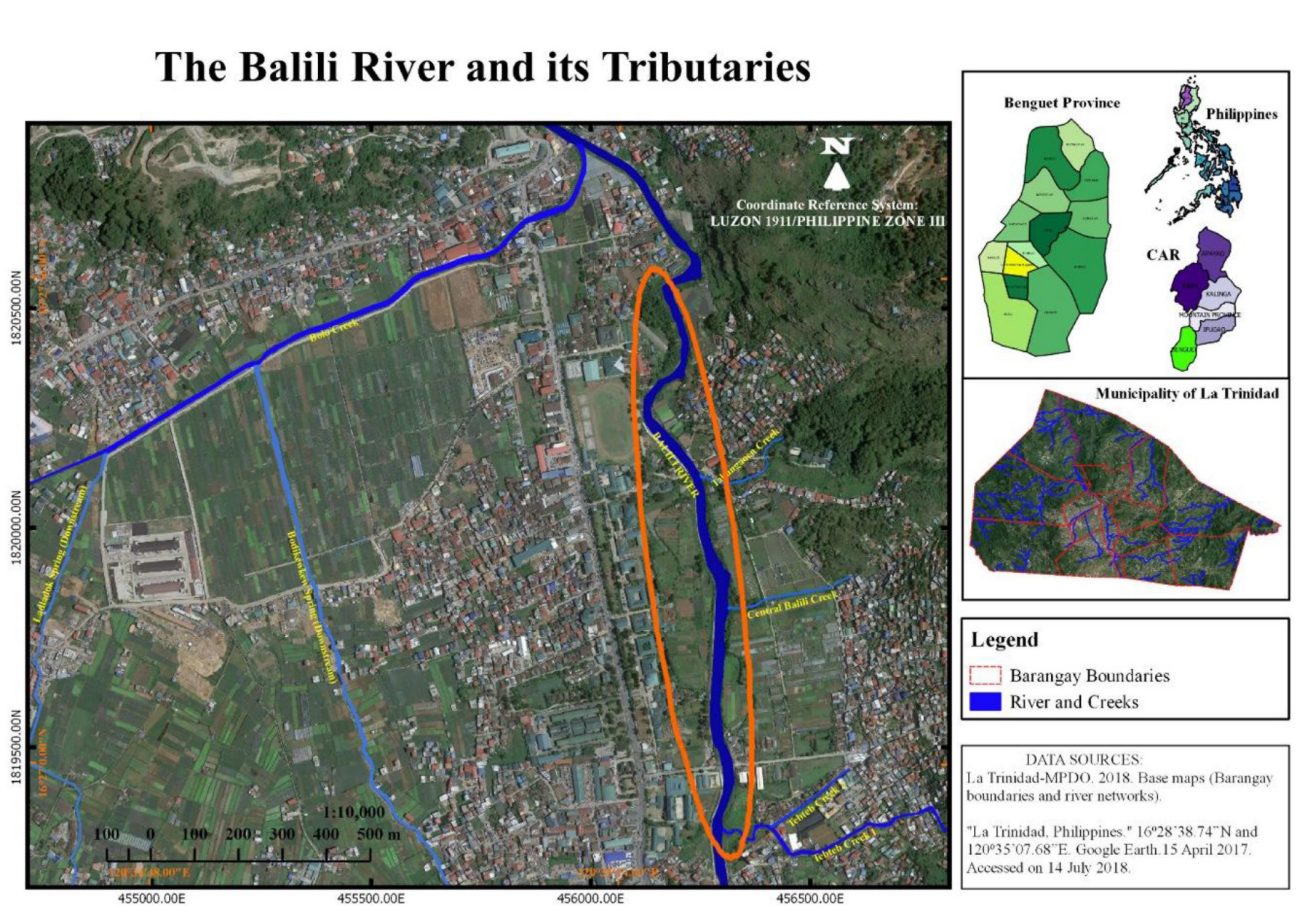
Balili River map showing the plant collection sites (in orange circle).

Balili River has a total length of 23.81 km traversing the city of Baguio, and the municipalities of La Trinidad and Sablan before entering the province of La Union (Fig. 1). The river suffers from excessive pollution which is usually blamed on the densely populated city of Baguio, its headwaters. The river was included in the DENR’s 2003 Pollution Report as one of the 15 “biologically dead” rivers among the 94 principal river basins in the country (Aro 2011; Palangchao 2011).

### Aquatic Macrophytes Used

Sampling stations were established along the main stream of Balili River in La Trinidad (from km. 5 to km. 6 stretch) where aquatic macrophytes were inventoried. The result of the inventory was reported in another study (Napaldet & Buot 2019). From this inventory, plants with phytoremediation potentials were identified. According to Moffat (1995) and US-EPA (1993), plants ideal for phytoremediation are species that have the ability to extract or degrade the contaminants, high adaptation to local climates, high biomass, deep root structure, compatibility with soils, high growth rate and easy to plant and maintain. Aquatic macrophytes in the area that fit these criteria were selected for the study, namely: *Amaranthus spinosus*, *Eleusine indica* and *Pennisetum purpureum*.

### Analysis of Hg and Pb

Plant individuals of *A. spinosus*, *E. indica* and *P. purpureum* were harvested along the littoral zone of the mainstream from km. 5 to km. 6 stretch of the river, selecting samples nearest to the water channel (see Fig. 1). Leaf, stem and root samples were randomly gathered from several plant individuals to derive a composite sample of at least 100g per replicate. Three replicates per plant organ were prepared, thus, each plant organs had a total of 300 g each. The samples were chopped into small sizes then submitted to Department of Science and Technology-Cordillera Administrative Region (DOST-CAR) laboratory for Pb and Hg determination. This laboratory analyses Pb by atomic atomic absorption spectrophometry after microwave digestion and Hg by thermal decomposition amalgamation and atomic absorption spectrophometry. It is an ISO-certified laboratory with an ISO No. 9001:2015. Quality control/assurance for all Hg and Pb analysis, including plants, soil and water, was based on the use of certified samples, samples from inter-laboratory comparisons, internal control samples and duplicates of the analysis.

Soil samples and water samples were also gathered from same area where the plant samples were collected. Soil samples were derived along the littoral zone at a 0–25 cm depth plant root zone (Baker *et al*. 1994; Gonzalez & Gonzalez-Chavez 2006) to form a composite sample of 1 kg each replicate. Three replicates per soil sample were prepared, thus, a total of 3 kg soil samples was submitted. The sample was then air-dried then submitted to same DOST-CAR laboratory for Pb and Hg determination. For water analysis of Pb and Hg, three replicates of 250 mL water samples were collected from the river and submitted for analysis in the same laboratory. Pb and Hg in water samples were compared with DENR Administrative Order (DAO) No. 2016-08 which sets the standard values for water quality monitoring in the Philippines.

### BCF and Translocation or Transfer Factor (TF) Determination

To evaluate the metal accumulation efficiency of each test plants, BCF and TF were calculated. BCF is defined as the ratio of the total metal concentration in the roots to that in the soil (Elkhatib *et al*. 2001; Gonzalez & Gonzalez-Chavez 2006; Yoon *et al*. 2006):

BCF=[M]roots/[M]soil

where [M]roots is the total metal concentration in the roots, and [M]soil is the total metal concentration in the soil, and wherein for this particular study the metal refers to Pb and Hg.

TF is defined as the ratio of the total Pb or Hg concentration in the shoots to the roots (Mocko & Waclawek 2004; Yoon *et al*. 2006; Sanghamitra *et al*. 2012). It was computed as follows:

TF=[M]leaves/[M]roots

where [M]leaves is the total Pb or Hg concentration in the leaves, and [M]roots is the total Pb or Hg concentration in the roots.

TF indicates internal metal transportation (Nouri *et al*. 2009). According to Yoon *et al*. (2006), both the BCF and TF can be used to estimate a plant’s potential for phytoremediation; BCF is used to estimate a plant’s ability to accumulate the metal in the roots while TF is used to estimate a plant’s ability to translocate metals from the roots to above-ground parts. Plants exhibiting BCF and TF values less than one are unsuitable for phytoextraction (Yoon *et al*. 2006). Plants with both BCF and TF greater than one (BCF > 1, TF > 1) have the potential to be used in phytoextraction. Besides, plants with bioaccumulation factor greater than one and translocation factor less than one (BCF > 1 and TF < 1) have the potential for phytostabilisation. A hyperaccumulator plant should have BCF > 1 or TF > 1, as well as total accumulation > 1000 mg kg^−1^ of Cu, Co, Cr or Pb, or > 10000 mg kg^−1^ of Fe, Mn or Zn (Kabata-Pendias 2011).

### Statistical Analysis

Data gathered on Pb and Hg concentrations in the different macrophytes and the different plant organs were subjected to appropriate statistical analyses such as Analysis of Variance (ANOVA), which determined significant differences between treatments, followed by Tukey’s HSD, as a post hoc Test.

## RESULTS

### Hg and Pb Concentration in Soil and Wastewater of Balili River

Hg in Balili River water samples registered very low values (0.000043 ug L^−1^) compared to the 2.0 ug L^−1^ standard set in DAO No. 2016-08. However, Pb in water registered values higher than the standard (2.36 vs. 0.01 mg L^−1^). This result was consistent with the water quality monitoring of DENR-CAR which showed that Balili River is contaminated with Pb but not with Hg (Napaldet & Buot, 2019). Pb levels in Balili River were comparable with usual urban run-off levels at 0.21–3.7 mg L^−1^ (European Commission 2001). This means that crops irrigated directly by untreated water from Balili River were not at risk of Hg contamination but were at risk for Pb.

On the other hand, the Hg concentrations in the soil/sediment (0.001 ug kg^−1^) were much higher than in the water, though still below the 1 mg kg^−1^ standard for soil (Environment Agency 2009). This was consistent with the conclusion of Weber *et al*. (2013) and Zhang *et al*. (2010a) that sediments contain higher heavy metal concentrations than the water. In Balili River, this was most likely due to retention of heavy metals by clay and organic particles in the sediment and, probably but minimally, thru enrichment directly from the weathering.

For Pb, soil concentrations were comparable with the water. But unlike water, Pb in soil concentrations in Balili River was way below common standards such as 100 mg kg^−1^ of Austria standard and 300 mg kg^−1^ of European Union standard (Maleki *et al*. 2014).

### Uptake of Hg and Pb in Local Aquatic Macrophytes

Aquatic macrophytes of Balili River differed in their Hg and Pb content, which indicated that they differed in their capacities for metal uptake, as observed also in other studies on metal contaminated sites (Freitas *et al*. 2004; Nouri *et al*. 2009; Nazareno & Buot 2015).

#### Hg

**Figure 2:**
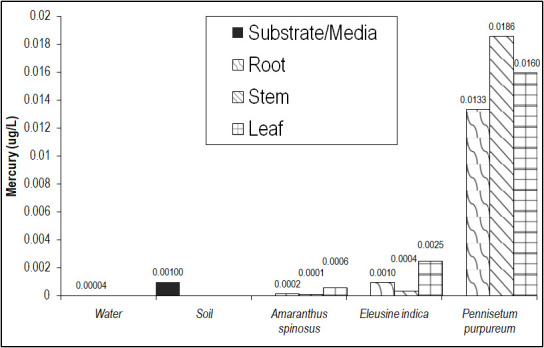
Hg concentration in major plant organs of the dominant macrophytes of Balili River showing highest accumulation in *P. purpureum*.

The Hg concentrations in major plants organs of the dominant macrophytes were presented in Fig. 2 and Table 1. It can be readily gleaned from the figure that Hg concentrations in plant tissues were significantly (*p* = 0.00) higher than in water and soil media. Among the macrophytes, *P. purpureum* recorded significantly higher (*p* = 0.00) Hg concentration in all of its major organs while least in *A. spinosus*. Consequently, *P. purpureum* also had the highest BCF and TF factor for Hg (Table 2). *P. purpureum* recorded a very high BCF of 13 and TF > 1 for Hg which qualifies this plant as an excellent Hg phytoextractor based from the criteria set in Kabata-Pendias (2011).

Results showed that *E. indica* and *A. spinosus* were both not good phytoaccumulator of Hg. However, both plants recorded high TF values on their leaves. This means that whatever little Hg these plants absorb, it is readily translocated and stored in their leaves. This contrasts with *P. purpureum* which stores more Hg in its stem. Nonetheless, both mechanisms are ecologically important since these plants absorb the Hg from the water and soil thus reducing its bioavailability and its threat to human health.

#### Pb

**Figure 3:**
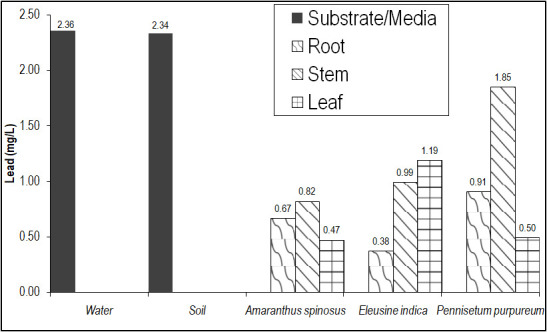
Pb concentration in major plant organs of the dominant macrophytes of Balili River.

*P. purpureum* also recorded the highest Pb concentrations except for leaf which was recorded in *E. indica* (Fig. 3). Consequently, *P. purpureum* have highest BCF for Pb while *E. indica* recorded the highest TF. Among the macrophytes, none recorded a BCF of >1 but *P. purpureum* and *E. indica* had TF of 2–3, which would qualify them as good phytoaccumulator. High TF denotes translocation of the metal from the roots to the shoots. This result showed that no macrophytes in the study would qualify as a hyperaccumulator of Pb.

## DISCUSSION

### Hg

The high translocation of Hg in shoots of the local aquatic macrophytes (leaf only in *E. indica* and *A. spinosus*) differs with other studies which generally found low translocation of Hg. The difference could be attributed to the low Hg concentration in the media of our study while much higher in the following studies. Minimal Hg translocation from roots to shoots was recorded in *Festuca rubra*, *Poa pratensis*, *Armoraci lapatifolia*, *Helianthus tuberosa* and *Salix viminalis* by Sas-Nowosielska *et al*. (2008), in *Vigna mungo* by Hussain *et al*. (2010) and in *Brassica juncea* by Moreno *et al*. (2008). Though the mechanism is not yet fully understood, the minimal translocation of Hg could be due to immobilisation by negatively charged pectins within the cell wall, precipitation of insoluble Pb salts in intercellular spaces, accumulation in plasma membranes, sequestration in the vacuoles of rhizodermal and cortical cells and physical barrier provided by the root endodermis (Pourrut *et al*. 2011).

Our result is more consistent with Rodriguez *et al*. (2005) which recorded significant translocation (TF > 1) of Hg in *Triticum aestivum*, *Hoduem vulgare* and *Lupinus luteus*. Also, significant translocation of Hg was recorded in *Pistia stratiotes* (Skinner *et al*. 2007) and in *Triticum durum* (Cavallini *et al*. 1999). In *Triticum durum*, Hg was found incorporated in leaf epidermal and stomatal cell walls and on parenchyma cell nuclei and this could likely be true in the local aquatic macrophytes of our study. However in *P. purpureum*, higher translocation of Hg was observed in the stem where it was likely isolated in the cells’ vacuole. This is could be attributed to the wide ground tissues in stem of the plant which consist of large parenchyma (large vacuoles). Cells’ vacuoles were determined a major storage site of heavy metals including Hg (US Department of Energy 1994).

The high BCF and TF values in *P. purpureum* make the plant ideal for phytoremediation of Hg that could be used in mining areas particularly in rivers that are recipient of small-scale mining activities in the region. Small scale mining activities in the region are not heavily monitored by DENR and do not have centralised tailing ponds so their tailings are directly dumped to nearby rivers. However, there’s a need to verify if this plant can duplicate its performance in higher Hg concentrations.

### Pb

Unlike in Hg, *E. indica* and *A. spinosus* had better performance in Pb uptake. *E. indica* recorded the highest concentration and TF of Pb in leaves (TF = 3.17). This showed *E. indica* as a good phytoaccumulator of Pb which agreed with the findings of Malik *et al*. (2010). However, other researchers found this plant inefficient for removal of other heavy metals such as As (Visoottiviseth *et al*. 2002), Cu, Cd, Cr and Co (Garba *et al*. 2012).

*A. spinosus*, on the other hand, had the same mechanism of Pb accumulation with *P. purpureum*, albeit in lower concentration. Highest concentration of Pb was recorded in the stem which is most likely stored in the parenchymatous cortex and pith. *A. spinosus* recorded a TF value of 1.23 for Pb which qualifies it as a phytoacumulator but was the lowest among the three macrophytes investigated. Nonetheless, this agreed with Chinmayee *et al*. (2012) that found this plant suitable not just for Pb accumulation but also for other heavy metals such as Cu, Zn, Cr and Cd.

In the three macrophytes in our study, the shoots have higher Pb concentration than the roots. The macrophytes show significant translocation (TF > 1) of Pb particularly in *P. purpureum* and *E. indica*. This result is significant since most plant species accumulated the absorbed Pb (approximately 95% or more) in their roots, and only a small fraction is translocated to aerial plant parts, as reported in *Vicia faba*, *Pisum sativum*, and *Phaseolus vulgaris*, *V. unguiculata*, *Nicotiana tabacum*, *Lathyrus sativus*, *Zea mays* and *Avicennia marina* (Pourrut *et al*. 2011). Thus, this study was able to discover new plants namely *P. purpureum* and *E. indica* that could be used in phytoremediation of Pb.

From these results, it was obvious that *P. purpureum* had the best potential for phytoremediation of Hg and Pb among the three macrophytes. It was an excellent phytoextractor of Hg (albeit in low concentration) and a good accumulator of Pb. It could be readily gleaned from Figs. 2 and 3 that the plant had higher Hg and Pb concentration in its shoot than in its roots signifying translocation of these metals after uptake by the roots. Also, the stem had significantly higher concentrations (*p* = 0.00) indicating that its heavy metal uptakes are primarily stored in the stem. This is readily conceivable because of the wide span of parenchymatous ground tissues in its long, numerous stems, which primarily function for storage of substances including heavy metals. Also, the high biomass and fast growth rate of the plant made it more ideal for phytoremediation. These findings add to the growing list of literature showing the phytoremediation potential of *P. purpureum*. Zhang *et al*. (2010b; 2014) found this plant to be a good phytoremediator of Cd, Zn and Cs while Silveira *et al*. (2013) documented it to be an excellent P remover.

The TF values for Hg in *P. purpureum*’s stem and leaves may be high but it was not believed to pose as health hazard even if this grass was regularly harvested as fodder for livestock. This is due to the low levels of absorbed Hg. Similarly, even if the Pb uptake and accumulation were higher, it was not believed to pose as health hazard since the recommended limits of lead in plants is as high as 10 ppm (Sadeghi *et al*. 2014). The same could be said for *E. indica* and possibly to other grasses in the littoral zone of Balili River that are being harvested as fodder.

## CONCLUSION

Soil and water samples of Balili River were found contaminated with Pb but not with Hg. Higher concentration of Hg was recorded in soil than water while Pb concentration did not differ between the two media. The aquatic macrophytes used in the study differed in their capacities for Hg and Pb uptake. Hg distribution follows this order: leaf > root > stem in *A. spinosus* and *E. indica* while stem > leaf > root in *P. purpureum*. On the other hand, Pb distribution follows this order: stem > root > leaf in *A. spinosus*; leaf > stem > root in *E. indica*; and, stem > root > leaf in *P. purpureum*. Highest concentration of Hg was recorded in *P. purpureum* across all major organs while least in *A. spinosus*. Same trend in Pb, *P. purpureum* recorded the highest concentrations except for leaf which was recorded in *E. indica*. Consequently, *P. purpureum* also had the highest BCF and TF factor for Hg and BCF for Pb while *E. indica* recorded the highest TF for Pb. *P. purpureum* recorded a very high BCF of 13 and TF > 1 for Hg which qualifies this plant as an excellent Hg phytoextractor. On the other hand, no macrophytes recorded a BCF of > 1 for Pb but *P. purpureum* and *E. indica* had TF of 2–3 which would qualify them as good phytoaccumulator. This result is significant since most plant species accumulated the absorbed Pb in their roots, and only a small fraction is translocated to aerial plant parts thus, *P. purpureum* and *E. indica* could be recommended for phytoremediation of Pb.

## Figures and Tables

**Table 1:** Difference in heavy metals concentration in major plant organs of the dominant macrophytes of Balili River.

Aquatic Macrophytes	Heavy metal concentration in major plant organs		

	Root	Stem	Leaf
(Hg)	μg/kg		
*A. spinosus*	0.00017^a^ ^I^	0.00013^a^ ^I^	0.00058^a^ ^I^
*E. indica*	0.00095^a^ ^I^	0.00037^a^ ^I^	0.00252^a^ ^I^
*P. purpureum*	0.01333^a^ ^II^	0.01857^a^ ^II^	0.01597^a^ ^II^

(Pb)	mg/kg		

*A. spinosus*	0.6700^a^ ^I^	0.8233^a^ ^I^	0.4733^a^ ^I^
*E. indica*	0.3767^a^ ^I^	0.9933^a^ ^I^	1.1933^a^ ^I^
*P. purpureum*	0.9100^ab^ ^I^	1.8533^b^ ^II^	0.4967^a^ ^I^

*Note:* Means with the same letter in a row are not significantly different at 0.05 Tukey’s HSD Mean with the same Roman number across column are not significantly different at 0.05 Tukey’s HSD

**Table 2:** BCF and TF of dominant macrophytes of Balili River for selected heavy metals.

Plant species	Hg			Pb		

	BCF	TF_stem_	TF_leaf_	BCF	TF_stem_	TF_leaf_
*A. spinosus*	0.166	0.780	3.500	0.286	1.229	0.706
*E. indica*	0.947	0.389	2.649	0.161	2.637	3.168
*P. purpureum*	13.289	1.393	1.198	0.389	2.037	0.546
